# The Influence of Obesity on Cholecystectomy Outcomes: A Systematic Review of Laparoscopic and Open Approaches

**DOI:** 10.7759/cureus.66171

**Published:** 2024-08-05

**Authors:** Nay Phone Hlyan, Lara Alsadoun, Muhammad Mustaneer Ul Hassan, Muhammad Junaid Cheema, Asghar Ali, Abdullah Shehryar, Abdur Rehman, Muhammad Usman Fareed

**Affiliations:** 1 General Surgery, Barts Health NHS Trust, London, GBR; 2 Trauma and Orthopaedics, Chelsea and Westminster Hospital, London, GBR; 3 General Surgery, Sir Ganga Ram Hospital, Lahore, PAK; 4 Internal Medicine, Allama Iqbal Medical College, Lahore, PAK; 5 Surgery, Mayo Hospital, Lahore, PAK; 6 Surgery, Nishtar Medical University, Multan, PAK

**Keywords:** complication rates, operative time, surgical outcomes, open cholecystectomy, laparoscopic cholecystectomy, obesity

## Abstract

This systematic review evaluates the impact of obesity on the outcomes of laparoscopic versus open cholecystectomy, analyzing data from five key studies. The review explores differences in operative times, complication rates, conversion rates, and recovery times among obese patients undergoing these surgical procedures. The findings indicate that while laparoscopic cholecystectomy in obese patients tends to require longer operative times, it does not significantly increase complication rates compared to open cholecystectomy. However, the risk of conversion to open surgery is modestly elevated. The review highlights the necessity for surgical guidelines to adapt to the challenges posed by obesity, recommending advanced training and innovative technologies to improve surgical outcomes. Limitations such as study design heterogeneity and variability in defining obesity underscore the need for further research. This review contributes to optimizing surgical care strategies and improving patient outcomes in the growing demographic of obese surgical patients.

## Introduction and background

Cholecystectomy, the surgical removal of the gallbladder, stands as a cornerstone in the management of gallbladder diseases, including symptomatic gallstones and cholecystitis [1]. With the advent of laparoscopic techniques in the late 20th century, laparoscopic cholecystectomy (LC) has become the gold standard due to its minimally invasive nature, offering reduced postoperative pain, shorter hospital stays, and quicker recovery times compared to the traditional open cholecystectomy (OC) [2]. However, the rise in global obesity rates presents new challenges in surgical management. Obesity increases the risk of gallbladder disease and complicates its surgical treatment due to factors like poor visualization, increased operative time, and a higher risk of complications, which can affect the choice and outcomes of surgical techniques [3].

The prevalence of obesity complicates the perioperative and postoperative landscape significantly. In obese patients, the increased visceral fat and a larger liver can obscure anatomical landmarks, making laparoscopic procedures technically challenging and increasing the risk of conversion to open surgery [4]. Furthermore, the demographic variations within the obese population, such as age and gender differences, can further influence surgical complexity and outcomes. For instance, older obese patients might face higher risks of complications due to decreased physiological reserve, and gender-specific fat distribution can affect the technical ease of the surgical procedure. Obese patients are also at a higher risk for surgical site infections, postoperative hernias, and prolonged recovery times. These challenges necessitate a deeper exploration of surgical outcomes in this demographic to optimize preoperative planning, surgical approach, and postoperative care [5]. This comprehensive approach will help tailor surgical strategies to individual patient profiles, enhancing safety and efficacy.

Despite the widespread adoption of laparoscopic methods, the debate between the benefits and limitations of LC versus OC in obese patients remains a significant point of contention in the surgical community [6]. This issue is compounded by varying definitions of obesity, differences in surgical skills, and the evolution of surgical technology, which influence the outcomes and recommendations in clinical practice [7].

The primary objective of this systematic review is to elucidate the impact of obesity on the outcomes of LC versus OC. This review aims to synthesize current evidence to compare the effectiveness, safety, and complication rates of laparoscopic and open approaches in obese patients undergoing cholecystectomy. By examining a range of outcomes, including operative time, conversion rates, postoperative complications, and long-term recovery, this study seeks to provide a comprehensive analysis that can guide surgeons in choosing the most appropriate surgical strategy for obese patients. Additionally, this review intends to highlight gaps in the current literature and suggest areas for future research, contributing to the ongoing improvement of surgical care in this challenging patient population.

## Review

Materials and methods

Search Strategy

Our search strategy, designed in accordance with the Preferred Reporting Items for Systematic Reviews and Meta-Analyses (PRISMA) guidelines, aimed to identify relevant literature on the impact of obesity on LC versus OC outcomes. We conducted comprehensive searches in key electronic databases, including PubMed, Medline, Embase, the Cochrane Library, and Scopus, covering literature from the inception of each database through June 2024. Keywords and Medical Subject Headings (MeSH) were used, incorporating terms such as "obesity," "laparoscopic cholecystectomy," "open cholecystectomy," and "surgical outcomes." Search queries were structured using Boolean operators to combine these terms effectively, for instance, "obesity AND laparoscopic cholecystectomy AND outcomes."

To ensure thoroughness, we also examined reference lists of selected articles and searched clinical trial registries and relevant conference proceedings to identify unpublished studies. The search was limited to English-language, peer-reviewed articles, focusing on clinical trials, observational studies, and meta-analyses that addressed surgical outcomes in obese patients undergoing these cholecystectomy techniques. This strategy provided a robust foundation for analyzing and synthesizing the most relevant and recent data.

Eligibility Criteria

The eligibility criteria for this systematic review were stringently defined to ensure the inclusion of relevant and high-quality studies analyzing the impact of obesity on the outcomes of LC versus OC. We focused exclusively on peer-reviewed research articles encompassing clinical trials, observational studies, and meta-analyses. To be included, studies must involve obese patients undergoing either LC or OC, with obesity defined according to standard BMI classifications or equivalent clinical criteria. The research must specifically report on operative time, complication rates, conversion rates, postoperative recovery, or long-term surgical outcomes.

Conversely, the exclusion criteria are designed to maintain the focus and quality of the review. We excluded studies that do not directly compare LC and OC outcomes in obese patients. Also excluded are case reports, editorials, review articles without meta-analyses, and studies focusing on non-obese populations. Research articles not written in English or published outside the window from the inception of each database until June 2024 are also omitted. This ensures that the review is current and relevant, adhering strictly to studies that meet rigorous academic standards and provide direct insights into the surgical management of obesity.

Data Extraction

Our data extraction process was carefully designed to ensure a thorough and accurate collection of data for our systematic review of the impact of obesity on outcomes of LC versus OC. Initially, articles were screened based on titles and abstracts to determine relevance. Two independent reviewers assessed each article, categorizing them as "relevant," "not relevant," or "potentially relevant." This preliminary filtering was crucial to identify the most pertinent studies for detailed analysis.

Following this initial screening, articles classified as potentially relevant were subjected to a full-text review. Data extraction was done using a standardized form in Microsoft Excel (Microsoft Corporation, Redmond, WA), ensuring uniformity across the process. The reviewers evaluated each article independently according to predefined inclusion and exclusion criteria. Discrepancies between reviewers were resolved through discussion, often involving a third senior reviewer. This systematic approach allowed for the precise extraction of key information, including study details, population characteristics, and surgical outcomes, facilitating a comprehensive synthesis of the findings.

Data Analysis and Synthesis

Given the diversity in study designs and outcomes, we opted for a qualitative rather than a quantitative meta-analysis approach to analyze the data from our systematic review on the impact of obesity on LC versus OC outcomes. This narrative synthesis method allowed us to delve deeply into the nuanced differences and extract meaningful insights across the studies, especially focusing on how obesity influences surgical outcomes.

We organized the data by categorizing key findings to discern common patterns and discrepancies related to surgical techniques and patient outcomes in obese populations. Through this thematic analysis, we identified critical factors influencing the effectiveness of LC and OC in obese patients, such as operative time, complication rates, and recovery trajectories. Our synthesis provided a comprehensive overview of the existing evidence, highlighting each surgical approach's relative advantages and challenges for obese patients. Additionally, we discussed the implications of these findings within the broader surgical context, pinpointed gaps in current research, and proposed directions for future studies. This structured synthesis clarified the relationships and contrasts among the studies and evaluated the evidence's robustness and applicability, contributing valuable perspectives on optimizing surgical care for obese patients.

Results

Study Selection Process

**Figure 1:**
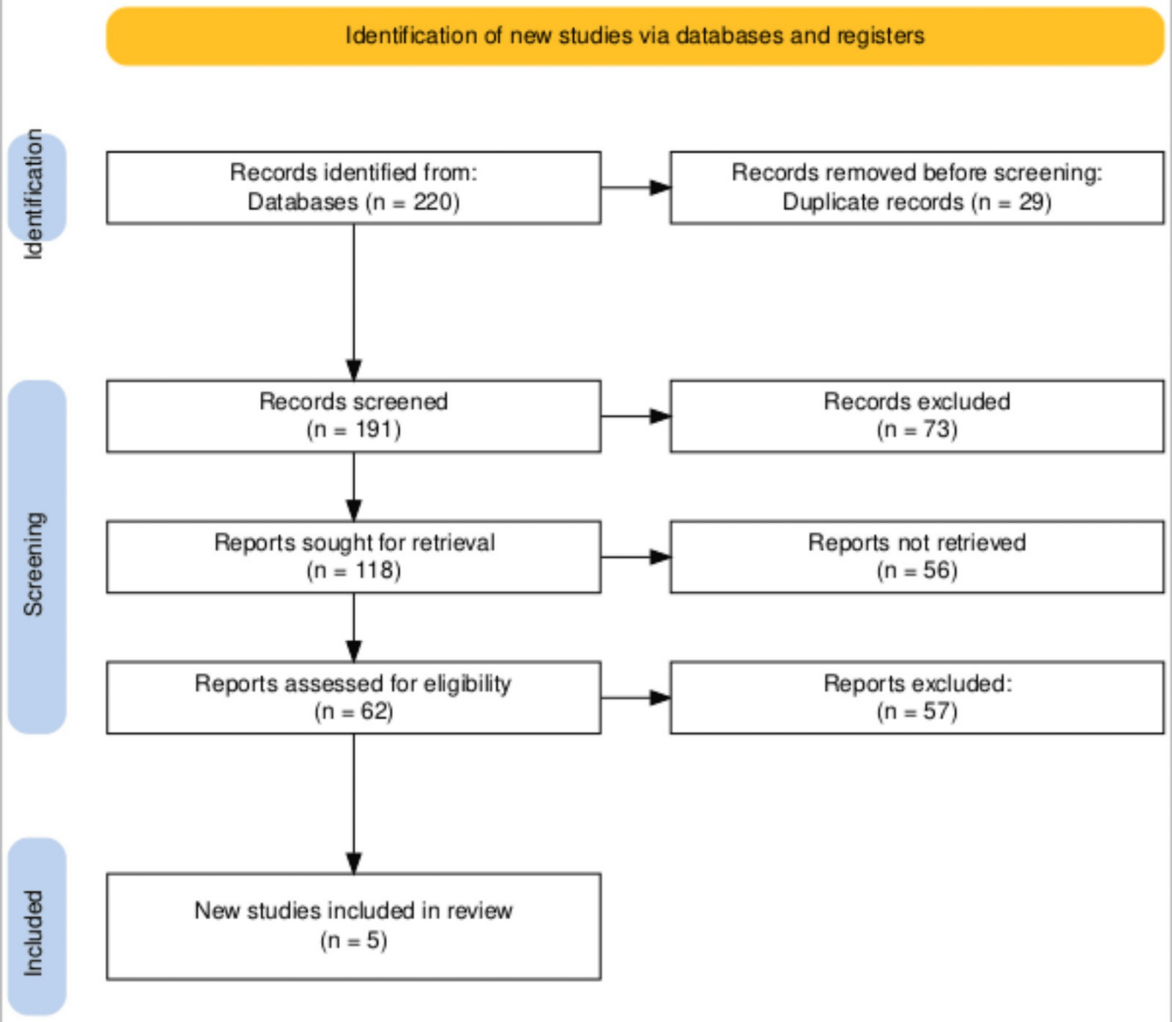
PRISMA flowchart representing the process of selection of studies. PRISMA: Preferred Reporting Items for Systematic Reviews and Meta-Analyses.

The search was conducted across multiple electronic databases, and initially, 220 records were identified. After removing 29 duplicate records, 191 were screened for relevance. This screening process led to the retrieval of 118 reports for more detailed assessment. Of these, 62 reports were further assessed for eligibility based on predefined inclusion and exclusion criteria. Ultimately, only five new studies met all the criteria and were included in our systematic review. This selection process ensures a rigorous and systematic literature review, adhering strictly to our research objectives and quality standards. Details of the process have been provided in Figure 1.

Characteristics of Selected Studies

The studies reviewed span a range of methodologies and focus areas within the context of LC among different patient demographics, primarily targeting obese patients. Habeeb et al. [8] conducted a multicenter randomized controlled trial to assess the safety and efficacy of concomitant LC with sleeve gastrectomy (SG) versus delayed LC post-SG in obese patients with asymptomatic gallbladder stones, involving 222 patients over a three-year period. Gatsoulis et al. [9] compared the performance of LC in obese versus nonobese patients in a one-year clinical trial involving 145 patients, noting no significant differences in hospital stays and similar complication rates across both groups. Harju et al. [10] evaluated the efficiency and recovery times between mini-cholecystectomy (MC) and LC, with their study showing that MC was faster without affecting outcomes related to hospital stay or complications. Burnand et al. [11] explored the impact of a very low-calorie diet (VLCD) on LC outcomes in obese patients, finding that preoperative weight loss led to shorter operative times and easier surgical procedures. Lastly, Hutchinson et al. [12] identified preoperative factors in 587 patients that predicted the need for conversion from LC to open surgery, highlighting the role of a thickened gallbladder wall and a dilated common bile duct as significant predictors. These studies collectively emphasize varied approaches to improving surgical outcomes and procedural efficiency in LC, particularly in relation to patient's body mass index (BMI) and preoperative conditions. These characteristics and key findings from each study are summarized briefly in Table 1.

**Table 1:** Summary of study characteristics and key findings on the impact of obesity on cholecystectomy outcomes. LC: laparoscopic cholecystectomy; SG: sleeve gastrectomy; BMI: body mass index; MC: mini-laparotomy cholecystectomy; VLCD: very low-calorie diet.

Authors	Study type	Background	Methods	Results	Conclusions
Habeeb et al. (2022) [8]	Randomized controlled trial	Evaluated safety and effects of concomitant LC with SG vs. delayed LC after SG in obese patients with asymptomatic gallbladder stones	Study period: January 2016 to January 2019; 222 morbidly obese patients; randomized: SG + LC vs. SG-only (111 each); multicenter study	LC added 40.7 minutes to SG conversion: 2.7% (SG+LC), 3.2% (delayed LC); complications: 9% (SG+LC) and 6.4% (delayed LC); 55% of SG-only needed later LC	Concomitant LC with SG is safe and prolongs operative time but minimizes the need for a second surgery
Gatsoulis et al. (1999) [9]	Clinical trial	Compared LC performance in obese and nonobese patients	Study period: November 1997 to November 1998; 145 patients (23 obese, 122 nonobese); comparative study	Operative time: 95 minutes (obese), 78 minutes (nonobese); conversion: 0% (obese) and 2.4% (nonobese); similar complication rates; no difference in hospital stay	LC is safe and effective for both obese and nonobese patients
Harju et al. (2006) [10]	Randomized controlled trial	Compared efficiency and recovery times of MC vs. LC, especially in obese patients	Study period: not specified (published 2006); 157 patients; randomized: MC (n = 85) vs. LC (n = 72)	Operating time: MC = 55 minutes and LC = 79 minutes; no differences in hospital stay, sick leave, pain, or complications; BMI did not affect outcomes	MC is faster than LC and equally suitable for obese patients
Burnand et al. (2016) [11]	Randomized controlled trial	Investigated effects of VLCD before LC in obese patients	Study period: not specified (published 2016); 46 obese patients; randomized: VLCD vs. normal diet for 2 weeks before LC	VLCD group: 3.48 kg weight loss; reduced operative time: 25 vs. 31 minutes; easier Calot's triangle dissection. No differences in postoperative outcomes	Two-week VLCD before LC is safe and reduces operative time in obese patients
Hutchinson et al. (1994) [12]	Clinical trial	Identified preoperative factors predicting conversion to open cholecystectomy	Study period: May 1990 to January 1993; 587 patients (526 with detailed ultrasound); retrospective analysis	Higher conversion in males and BMI > 27.2 kg/m². Thickened gallbladder wall: 6x higher conversion rate; dilated common bile duct correlated with positive cholangiogram	Thickened gallbladder wall and dilated common bile duct predict higher conversion rates

Discussion

Our systematic review critically focuses on the impact of obesity on surgical outcomes, particularly in LC versus OC. The inherent challenges posed by obesity, such as increased abdominal fat and larger liver size, can complicate laparoscopic procedures and potentially affect various surgical outcomes, including operative times, complication rates, conversion rates, and recovery times [13].

Obesity has been consistently shown to increase operative times in laparoscopic surgeries. Habeeb et al. (2022) [8] found that LC added approximately 40.7 minutes to sleeve gastrectomy procedures in obese patients compared to those undergoing surgery without concomitant cholecystectomy. This indicates the additional care and caution required to maneuver within a limited visual field and space due to increased adiposity. The extended duration can be attributed to the difficulties in managing the enlarged fatty liver and navigating through thicker layers of adipose tissue, which are less of an issue in OC.

While obesity is associated with higher complication rates in general surgical procedures, our review indicates that this may not significantly differ between laparoscopic and open approaches in cholecystectomy. For instance, Gatsoulis et al. (1999) [9] reported similar complication rates between obese and nonobese patients undergoing LC, suggesting that with adequate experience and technique, laparoscopic methods can be equally safe. This finding challenges the traditional concerns regarding laparoscopic procedures in obese populations and underscores the importance of surgical expertise.

The conversion rate from LC to OC remains a pivotal concern in obese patients. According to Hutchinson et al. (1994) [12], specific preoperative factors such as a thickened gallbladder wall significantly increase the likelihood of conversion. This is critical as it highlights the need for preoperative imaging and evaluation to better prepare for potential intraoperative challenges. Although laparoscopic techniques are preferred for their minimally invasive nature, these findings suggest a heightened readiness for conversion in obese patients should be part of surgical planning.

The benefits of LC in terms of recovery times are well documented; however, in obese patients, these advantages must be weighed against the increased risk of complications such as hernias and wound infections, which can prolong recovery. Harju et al. (2006) [10] found no significant differences in hospital stay or sick leave between mini-laparotomy and traditional LC, indicating that when laparoscopic surgery is feasible, it can offer similar recovery benefits to obese patients as it does to the general population [14].

Obesity introduces significant physiological and anatomical challenges that can complicate laparoscopic surgical procedures, impacting both surgical decisions and outcomes [15]. The increased abdominal fat in obese patients can reduce visibility and limit maneuverability within the surgical field, posing substantial risks during procedures like LC [16]. These issues are compounded by the more significant liver often seen in obese patients, which can obscure the gallbladder and make access more difficult. Consequently, these factors contribute to a higher conversion rate from laparoscopic to open surgery as surgeons may opt for an open approach when encountering unmanageable difficulties or risks that compromise patient safety during laparoscopic procedures [17].

The findings of this review have practical implications for surgical practice, especially concerning the management of obese patients undergoing cholecystectomy. Surgeons can use these data to enhance preoperative planning by incorporating routine assessments of patient-specific anatomical challenges posed by obesity [18]. Additionally, understanding the increased risks associated with laparoscopic procedures in this population could guide patient counseling on surgical risks and recovery expectations [19]. Surgeons might consider modifying traditional laparoscopic techniques or employing advanced technologies such as high-definition cameras or robotic assistance to improve visibility and precision during surgeries in obese patients, potentially reducing the need for conversion to open procedures and improving overall surgical outcomes [20].

The current literature, while extensive, reveals several gaps, particularly in the areas of long-term outcomes and specific complications associated with cholecystectomy in obese patients [21]. Studies often focus on immediate or short-term surgical outcomes, leaving a gap in understanding the long-term effects of LC versus OC in this population. Additionally, data on specific complications, such as surgical site infections and their management in obese patients, are limited [22]. Future research could address these gaps by designing longitudinal studies that follow obese patients over extended periods to evaluate long-term surgical outcomes and complications. Further research could also explore the effectiveness of different prophylactic measures and surgical techniques tailored to reduce complication rates in obese individuals undergoing cholecystectomy [23].

The findings from this review suggest several updates to clinical guidelines for the management of cholecystectomy in obese patients. Guidelines should incorporate specific considerations for preoperative assessment focusing on anatomical variations in obese individuals that may affect surgical approach and outcomes. Enhanced imaging protocols and tailored surgical techniques, possibly including the use of advanced laparoscopic or robotic tools, should be recommended to address the unique challenges of operating on obese patients [24]. Concurrently, potential biases such as the selection of participants with differing degrees of obesity and the retrospective nature of some studies could affect the generalizability of the findings. Future guidelines must consider these limitations and the varying quality of evidence, predominantly from observational studies rather than randomized controlled trials.

Future research should aim to fill the existing gaps by conducting long-term prospective studies that compare LC versus OC outcomes across a more diverse obese population. This would help to establish more precise benchmarks for recovery, complication rates, and long-term effectiveness tailored to varying degrees of obesity. Additionally, exploring the impact of emerging surgical technologies, such as enhanced visualization systems and robotic assistance, could provide valuable insights into improving surgical safety and reducing conversion rates. Studies that assess the economic impact of different surgical approaches in obese patients could further support guideline updates and clinical decision-making, ensuring optimal patient-centered care [25].

Enhancing surgical education and training is crucial for improving outcomes in obese patients undergoing cholecystectomy. Simulation training and specialized modules focused on managing obesity during surgical procedures can equip surgeons with the skills and confidence to handle the anatomical and physiological challenges presented by obese patients [26]. Additionally, integrating multidisciplinary approaches involving dietitians, physiotherapists, and obesity specialists can enhance both preoperative and postoperative care, optimizing overall patient outcomes. Economic evaluations should complement these efforts to assess the cost-effectiveness of various surgical approaches, including potential differences in hospital stay durations, reoperation rates, and overall healthcare costs [27]. Such data are essential for informing health policy changes, advocating for resource allocation, and adjusting clinical practices to provide cost-effective and high-quality care for the obese population undergoing cholecystectomy [28].

In acknowledging the limitations of our systematic review, we recognize the importance of the risk of bias assessment for providing a more comprehensive understanding of the quality and reliability of the evidence presented. While our initial approach prioritized a qualitative synthesis of outcomes from various studies, the inclusion of a bias assessment could further elucidate the strength of these findings. Therefore, we have undertaken a preliminary assessment of potential biases inherent in the studies reviewed. Common issues identified include selection bias due to the non-randomized nature of many included studies, and reporting bias, as studies may have variably disclosed adverse outcomes. This discussion aims to guide readers in critically evaluating the implications of these biases on the reported results and should be considered when interpreting the effectiveness and safety of surgical options for obese patients undergoing cholecystectomy. Future updates to this review will aim to incorporate a more structured risk of bias analysis, which will enhance the replicability and validity of our findings, contributing to a more robust guideline for clinical practice.

## Conclusions

This systematic review critically evaluates the impact of obesity on the outcomes of LC versus OC, highlighting the adaptability and safety of both surgical approaches in obese patients when appropriate techniques are employed. Our findings advocate for refining surgical guidelines to better address the specific challenges posed by obesity, including enhanced preoperative planning, advanced surgical training, and the integration of multidisciplinary care teams. Furthermore, the review calls for ongoing research to address gaps in long-term outcomes and economic analyses, essential for developing comprehensive, cost-effective strategies tailored to the obese population. Ultimately, this work underscores the necessity of evolving surgical practices and health policies to improve the quality of care for obese patients undergoing cholecystectomy.
